# Predictive Value of Echocardiographic Strain for Myocardial Fibrosis and Adverse Outcomes in Autoimmune Diseases

**DOI:** 10.3389/fcvm.2022.836942

**Published:** 2022-02-21

**Authors:** Fuwei Jia, Xiao Li, Dingding Zhang, Shu Jiang, Jie Yin, Xiaojin Feng, Yanlin Zhu, Yingxian Liu, Yuanyuan Zhu, Jinzhi Lai, Huaxia Yang, Ligang Fang, Wei Chen, Yining Wang

**Affiliations:** ^1^Department of Cardiology, Peking Union Medical College Hospital, Peking Union Medical College and Chinese Academy of Medical Sciences, Beijing, China; ^2^Department of Radiology, Peking Union Medical College Hospital, Peking Union Medical College and Chinese Academy of Medical Sciences, Beijing, China; ^3^Medical Research Center, Peking Union Medical College Hospital, Peking Union Medical College and Chinese Academy of Medical Sciences, Beijing, China; ^4^Department of Rheumatology and Clinical Immunology, Peking Union Medical College Hospital, Peking Union Medical College and Chinese Academy of Medical Sciences, Beijing, China

**Keywords:** autoimmune diseases, global longitudinal strain, late gadolinium enhancement, myocardial fibrosis, outcome

## Abstract

**Background:**

Myocardial fibrosis is an important pathophysiologic mechanism of cardiac involvement that leads to increased mortality in patients with autoimmune diseases (AIDs). The aim of this study was to evaluate the association between myocardial strain from speckle-tracking echocardiography (STE) and fibrosis on cardiovascular magnetic resonance (CMR) and to further explore their prognostic implications in patients with AIDs.

**Methods:**

We prospectively included 102 AIDs patients with clinically suspected cardiac involvement and 102 age- and sex-matched healthy individuals. Patients underwent CMR for evaluation of myocardial fibrosis by late gadolinium enhancement (LGE) and T1 mapping. A semiquantitative evaluation based on the extent of LGE was used to calculate the total (tLGEs) and segmental (sLGEs) LGE score. Global longitudinal strain (GLS) was evaluated by STE in all subjects. All patients were regularly followed up every 6 months. The primary endpoint was the composite incidence of all-cause death and cardiovascular hospitalization.

**Results:**

Compared to healthy controls, AIDs patients had impaired GLS (−17.9 ± 5.1% vs. −21.2 ± 2.5%, *p* < 0.001). LGE was detected in 70% of patients. Patients with LGE presented worse GLS (−17.1 ± 5.3% vs. −19.6 ± 4.1%, *p* = 0.018) than those without LGE. On multivariate logistic analysis, GLS ≥ −15% was an independent predictor of LGE presence (OR = 4.98, 95%CI 1.35–18.33, *p* = 0.016). Moreover, a marked and stepwise impairment of segmental longitudinal strain (−19.3 ± 6.6 vs. −14.9 ± 6.5 vs. −8.9 ± 6.3, *p* < 0.001) was observed as sLGEs increased. During a median follow-up time of 25 months, 6 patients died, and 14 patients were hospitalized for cardiovascular reasons. Both GLS ≥ −15% (HR 3.56, 95%CI 1.28–9.86, *p* = 0.015) and tLGEs ≥ 6 (HR 4.13, 95%CI 1.43–11.92, *p* = 0.009) were independently associated with the primary endpoint.

**Conclusions:**

In AIDs patients, impaired myocardial strain on STE could reflect the presence and extent of myocardial fibrosis and provide incremental prognostic value in addition to LGE in the prediction of adverse outcomes.

## Introduction

Cardiac involvement is a common comorbidity of autoimmune diseases (AIDs) and leads to increased mortality not sufficiently explained by traditional cardiovascular risk factors (1–3). Myocardial fibrosis plays an important role in pathophysiologic mechanisms of cardiac impairment, as a result of autoimmune process and chronic inflammatory status commonly seen in AIDs (2, 4). Indeed, histopathological studies have demonstrated that myocardial fibrosis is prevalent in AIDs and indicates impaired cardiac function (2, 5). For instance, a high frequency (90%) of interstitial fibrosis in myocardium occurred in patients with systemic lupus erythematosus (SLE) based on endomyocardial biopsy (6). Although endomyocardial biopsy is the gold standard for the diagnosis of myocardial involvement, its invasive properties and complications sometimes limit its clinical application. Recently, late gadolinium enhancement (LGE) and T1 mapping on cardiovascular magnetic resonance (CMR) imaging have emerged as a well-established technique for detecting myocardial fibrosis in AIDs (7). Nearly half (46%) of rheumatoid arthritis (RA) patients exhibited focal fibrosis on LGE, and increased extracellular volume (ECV) is associated with disease activity (8). Moreover, global longitudinal strain (GLS) derived from speckle-tracking echocardiography (STE) has the potential to assess myocardial dysfunction in a timely manner (9). GLS has been reported to be up to 2% lower in AIDs patients with normal left ventricular ejection fraction (LVEF) than in the general population (10). However, whether impaired strain is associated with myocardial fibrosis in AIDs remains unknown. Furthermore, despite the potential utility of the above imaging parameters in identifying cardiac involvement, the prognostic significance of myocardial impairment evidenced by imaging has not been well-studied (11). In this regard, our study aimed to assess imaging evidence of myocardial involvement in AIDs patients with clinically suspected cardiac involvement, explore the relationship of myocardial strain and fibrosis, and search for potential risk factors to predict prognosis.

## Methods

### Study Population

Between 2011 and 2021, a prospective cohort of AIDs patients at the Peking Union Medical College Hospital was consecutively enrolled according to the following inclusion criteria: First, AIDs patients fulfilled the classification criteria of SLE (ACR 1997 criteria) (12), idiopathic inflammatory myopathy (IIM) (Bohan and Peter criteria) (13), Sjogren's syndrome (SS) (AECG 2002 criteria) (14), and SSc (ACR 1980 criteria) (15); Second, patients with clinically suspected cardiac involvement who met indications for CMR, including new onset or persisting cardiac symptoms (dyspnea, palpitations or chest pain), electrocardiogram (ECG) abnormalities, or elevated troponin (16, 17); Last, the interval of echocardiography and CMR imaging examinations was within 10 days. The exclusion criteria were as follows: age <18 years, estimated glomerular filtration rate (eGFR) <30 ml/(min·1.73 m^2^), congenital heart disease, ischemic heart disease, history of thoracic surgery and inadequate imaging quality. All patients underwent echocardiographic and CMR evaluations based on clinical indications.

Additionally, 102 age- and sex-matched healthy individuals were recruited as the control group and underwent an echocardiographic examination. Our study was registered at ClinicalTrials.gov (NCT03885375) and approved by the Institutional Review Board of Peking Union Medical College Hospital (no. ZS-1790). All participants provided written informed consent before our investigation.

### Echocardiography and Myocardial Deformation

All echocardiographic examinations were performed using an ultrasound system (Vivid 9, GE Medical System, Milwaukee, Wisconsin). Images were obtained by the recording of at least 3 consecutive cardiac cycles at a frame rate of ≥60 frames/s. Cardiac structure and function were measured in accordance with American Society of Echocardiography recommendations (18). We calculated LVEF using Simpson's method in the apical 2- and 4-chamber planes. Mitral inflow early (E) and late (A) peak velocities were measured via pulsed-wave Doppler. Tissue doppler imaging was used to measure mitral annular velocities (lateral e' and septal e'). Pulmonary artery systolic pressure was estimated based on tricuspid regurgitation velocity. Left atrial (LA) volume was measured using the biplane area-length method. LV diastolic dysfunction was assessed according to diagnostic criteria of the ASE/EACVI guidelines (19).

Myocardial deformation was analyzed via EchoPAC software (version 113, GE Medical Systems, Milwaukee, Wisconsin) as previously described (10). GLS, global circumferential strain (GCS) and global radial strain (GRS) during the systolic phase were acquired by averaging the strain of 17 segments. Low negative values of GLS and GCS reflected diminished myocardial contractility (20). Peak left atrial longitudinal strain (PALS) was defined as the peak positive longitudinal strain during the LA reservoir phase and was calculated by averaging the peak values of all LA segments (21). Two researchers independently performed the measurements and were blinded to patients' group and outcome.

### CMR Images and Analysis

CMR images were acquired using a 3.0 T scanner (MAGNETOM Skyra, Siemens Healthineers, Erlangen, Germany). We obtained cine images via an ECG-gated 2D balanced steady-state free precession sequence. LGE images were collected 10 min after the injection of 0.1 mmol/kg gadopentetate dimeglumine using a 2D phase-sensitive inversion-recovery gradient-echo pulse sequence. Native and postcontrast T1 mapping was performed using a modified look-locker inversion recovery sequence in a 4-chamber long-axis slice and apical, middle, and basal short-axis slices (22).

CMR images were independently analyzed by two experienced investigators blinded to STE and clinical outcome data. Visual assessment of LGE was performed according to standardized postprocessing recommendations (23). After qualitative analysis of the presence or absence of LGE, we also applied a semiquantitative evaluation to calculate segmental LGE score (sLGEs) based on the extent of LGE: no LGE involvement of the segment = 0, 1 to 50% LGE involvement of the segment = 1, and 51 to 100% LGE involvement of the segment = 2. Therefore, the total LGE score (tLGEs) of 17 segments ranged from 0 to 34 (24). Native T1 and ECV were analyzed semiautomatically via cvi42 software (version 5.3, Circle Cardiovascular Imaging, Calgary, Canada). ECV was calculated using the T1 of myocardium and T1 of blood pool pre- and post-gadolinium contrast, along with the hematocrit value (25).

### Endpoint and Follow-Up

The primary endpoint of our study was the composite incidence of all-cause death and cardiovascular hospitalization. We obtained follow-up information every 6 months through regular outpatient visits or telephone interviews.

### Statistical Analysis

Data are described as the mean ± SD, median (interquartile range [IQR]) or proportion, as appropriate. The χ^2^ test and Student's *t*-test were used to compare differences between two groups for categorical variables and continuous variables, respectively. Receiver operating characteristics (ROC) curves were created to identify thresholds for grouped variables. Univariate and multivariate logistic regression analyses were performed to determine the predictive value of clinical and echocardiographic variables for LGE presence. Univariate and multivariate Cox proportional hazards analyses were adopted to identify the predictors associated with adverse clinical outcomes. The initial univariate analyses were performed on the primary parameters of interest, including age, age at diagnosis of AIDs, sex, disease duration, NTproBNP, CTnI, LVEF, GLS, PALS, LGE presence and tLGEs. Subsequently, the variables with *p* < 0.05 in univariate analysis as well as age were included in multivariate analysis using a stepwise forward selection approach. Kaplan-Meier plots were constructed to display the event-free survival of subgroups according to GLS and tLGEs. Statistical analysis was performed using SPSS software (version 23, IBM, Chicago). A 2-tailed p < 0.05 was considered to indicate statistical significance.

## Results

### Clinical and Imaging Characteristics

A total of 116 AIDs patients with clinically suspected cardiac involvement were screened (Supplementary Figure 1). Of them, 14 patients were excluded due to age <18 years (*n* = 2), eGFR <30 ml/(min·1.73m^2^) (*n* = 1), ischemic heart disease (*n* = 2), or inadequate imaging quality (*n* = 9). Therefore, 102 AIDs patients were ultimately enrolled and followed up, including 48 with SLE, 38 with IIM, 5 with SS and 11 with SSc. All patients had complete images for STE and LGE analyses, and CMR images of 90 patients were available for the analysis of T1 mapping. Among the 102 enrolled patients, 14 were male and 88 were female, with a median disease duration of 21 (IQR, 4–84) months. The mean age was 35.8 ± 14.7 years at diagnosis of AIDs. Of all patients, 25 (25%) had NYHA class ≥ 2, 47 (46%) had ECG abnormalities, and 39 (38%) had cardiac troponin I (CTnI) elevation. Musculoskeletal (55%) and Mucocutaneous (37%) systems were the most common multi-organ involvements (Table 1).

**Table 1:** Baseline demographic and clinical characteristics of AIDs patients.

**Variables**	**AIDs patients (*n* = 102)**
Age (years)	40.0 ± 15.3
Age at diagnosis of AIDs (years)	35.8 ± 14.7
Female [n (%)]	88 (86%)
Body surface area (m^2^)	1.59 ± 0.17
Disease duration (months) [median (IQR)]	21 (4–84)
Treatment-naïve patients [*n* (%)]	42 (41%)
hsCRP (mg/L)	1.93 (0.78–6.93)
ESR (mm/h)	14 (8–33)
eGFR [ml/(min·1.73 m^2^)]	111 (88–148)
**Clinically suspected cardiac involvement [*****n*** **(%)]**
Symptoms^a^	30 (29%)
NYHA class≥ 2	25 (25%)
ECG abnormalities^b^	47 (46%)
CTnI elevation (> 0.056 ug/L)^c^	39 (38%)
**Multi-organ involvements [*****n*** **(%)]**
Musculoskeletal system	56 (55%)
Respiratory system	35 (34%)
Hematologic system	28 (27%)
Digestive system	3 (3%)
Mucocutaneous system	38 (37%)
Urinary system	26 (25%)
Nervous system	7 (7%)
**Comorbidity [*****n*** **(%)]**
Hypertension	23 (23%)
Diabetes	3 (3%)
CHD	12 (12%)
**Medications [*****n*** **(%)]**
Glucocorticoid	86 (84%)
Immunosuppressant^d^	68 (67%)
Beta-blockers	33 (32%)
ACE inhibitors/ARB	18 (18%)
Calcium antagonists	10 (10%)
Diuretics	43 (42%)

*ACE, angiotensin-converting enzyme; AIDs, autoimmune diseases; ARB, angiotensin T1 receptor blockers; CHD, coronary heart disease; CTnI, cardiac troponin I; ECG, electrocardiogram; eGFR, estimated glomerular filtration rate; ESR, erythrocyte sedimentation rate; hsCRP, hypersensitive C-reactive protein; IQR, interquartile range; NYHA class, New York Heart Association function classification*.

a *New onset or persisting symptoms suggestive of myocardial involvement: dyspnea, orthopnea, palpitations, effort intolerance/malaise or chest pain*.

b *Atrioventricular block I-III, bundle branch block, extrasystoles, supraventricular tachycardia, atrial fibrillation, ventricular tachycardia, ventricular fibrillation or ST-segment and T-wave changes*.

c *An elevated CTnI value above the 99th percentile upper reference limit*.

d *Azathioprine, cyclophosphamide, mycophenolate, cyclosporine, methotrexate, leflunomide, rapamycin or mycophenolate mofetil*.

Moreover, 102 healthy individuals (mean age 37.5 ± 13.8 years, 79% female) were included in our study. Compared to controls, AIDs patients were more likely to have impaired GLS (−17.9 ± 5.1% vs. −21.2 ± 2.5%, *p* < 0.001) and PALS (20.9 ± 11.7% vs. 32.5 ± 9.9%, *p* < 0.001) (Table 2). A quarter of AIDs patients had LV diastolic dysfunction. CMR revealed that 70% of patients presented LGE with a non-ischemic pattern: 21% only in the septum, 7% only in the free wall, and 43% in both locations. The median tLGEs was 4 (0–8). Among the 90 patients whose T1 mapping was available, native T1 and ECV were 1,390 ± 75 ms and 33.2 ± 6.0%, respectively, which were significantly higher than normal values obtained from healthy controls in our hospital (shown in Table 2). In addition, echocardiographic and CMR parameters did not differ significantly among the four types of AIDs (Supplementary Table 1). Similarly, no significant difference in the above parameters was found according to disease activity (Supplementary Table 2).

**Table 2:** Comparison of clinical and imaging parameters of study population.

**Variables**	**Healthy controls (*n* = 102)**	**All patients (*n* = 102)**	*p* ^*^	**LGE (–) (*n* = 31)**	**LGE (+) (*n* = 71)**	*p* ^**^
**Clinical variables**
Age (years)	37.5 ± 13.8	40.0 ± 15.3	0.160	39.1 ± 15.1	40.4 ± 15.4	0.785
Female [*n* (%)]	81 (79%)	88 (86%)	0.265	29 (94%)	59 (83%)	0.158
Disease duration (months) [median (IQR)]	–	21 (4–84)	–	14 (5–72)	24 (4–86)	0.693
NYHA class ≥ 2 [*n* (%)]	0 (0%)	25 (25%)	<0.001	3 (10%)	22 (31%)	0.025
ECG abnormalities [*n* (%)]	0 (0%)	47 (46%)	<0.001	10 (32%)	37 (52%)	0.085
CTnI elevation (>0.056 ug/L) [n (%)]	0 (0%)	39 (38%)	<0.001	7 (23%)	32 (45%)	0.045
**Echocardiography**
LVEF (%)	67.8 ± 6.4	60.5 ± 13.1	<0.001	64.3 ± 10.1	58.9 ± 14.0	0.056
LVEDVi (ml/m^2^)	49.6 ± 9.6	54.6 ± 18.1	0.135	51.7 ± 12.0	55.9 ± 20.2	0.497
LVESVi (ml/m^2^)	15.2 ± 4.2	22.7 ± 13.1	<0.001	18.6 ± 7.7	24.5 ± 14.5	0.072
E/A ratio	1.5 ± 0.4	1.2 ± 0.4	<0.001	1.2 ± 0.4	1.2 ± 0.4	0.597
Lateral e' (cm/s)	14.6 ± 3.6	9.6 ± 3.9	<0.001	10.2 ± 4.0	9.4 ± 3.8	0.477
Septal e' (cm/s)	11.0 ± 2.8	6.9 ± 2.6	<0.001	7.6 ± 2.8	6.6 ± 2.5	0.198
E/e'	6.8 ± 2.1	11.0 ± 6.0	<0.001	9.9 ± 4.6	11.5 ± 6.5	0.131
TRV (m/s)	2.1 ± 0.3	2.7 ± 0.7	<0.001	2.5 ± 0.6	2.7 ± 0.8	0.109
LAVi (ml/m^2^)	24.6 ± 7.1	33.5 ± 14.0	<0.001	32.5 ± 15.0	34.0 ± 13.6	0.397
PASP (mmHg)	22.9 ± 4.8	35.0 ± 19.5	<0.001	30.4 ± 15.2	36.9 ± 20.9	0.064
Diastolic dysfunction [*n* (%)]	0 (0%)	27 (26%)	<0.001	5 (16%)	22 (31%)	0.147
GLS (%)	−21.2 ± 2.5	−17.9 ± 5.1	<0.001	−19.6 ± 4.1	−17.1 ± 5.3	0.018
GCS (%)	−25.2 ± 4.4	−23.5 ± 6.7	0.043	−25.8 ± 7.0	−22.5 ± 6.4	0.021
GRS (%)	25.8 ± 11.8	18.2 ± 6.5	<0.001	18.6 ± 6.0	18.0 ± 6.7	0.551
PALS (%)	32.5 ± 9.9	20.9 ± 11.7	<0.001	23.6 ± 10.6	19.7 ± 12.0	0.126
**CMR**
LGE (+) [*n* (%)]	–	71 (70%)	–	0 (0%)	71 (100%)	<0.001
tLGEs	–	4 (0–8)	–	0	5 (4–11)	<0.001
Native T1 (ms) ^a^	1,268 ± 36 ^b^	1,390 ± 75	<0.001	1,344 ± 60	1,408 ± 74	<0.001
ECV (%) ^a^	26.2 ± 2.7 ^b^	33.2 ± 6.0	<0.001	31.5 ± 4.0	33.9 ± 6.5	0.205

*CMR, Cardiac magnetic resonance imaging; ECV, extracellular volume; EDV, end-diastolic volume; ESV, end-systolic volume; EF, ejection fraction; GCS, global circumferential strain; GLS, global longitudinal strain; GRS, global radial strain; LAVi, left atrium volume index; LGE, late gadolinium enhancement; LV, left ventricular; PALS, peak left atrial longitudinal strain; PASP, pulmonary artery systolic pressure; tLGEs, total LGE score; TRV, tricuspid regurgitation velocity. Other abbreviations as Table 1*.

* *Healthy controls vs. All patients*.

** *LGE (–) vs. LGE (+)*.

a *Analysis of T1 mapping was available for 90 AIDs patients*.

b *Normal values were obtained from 16 normal control subjects (49.1 ± 6.2 years, 50% female) in our hospital*.

### Relationships Between Strain and Fibrosis

**Figure 1:**
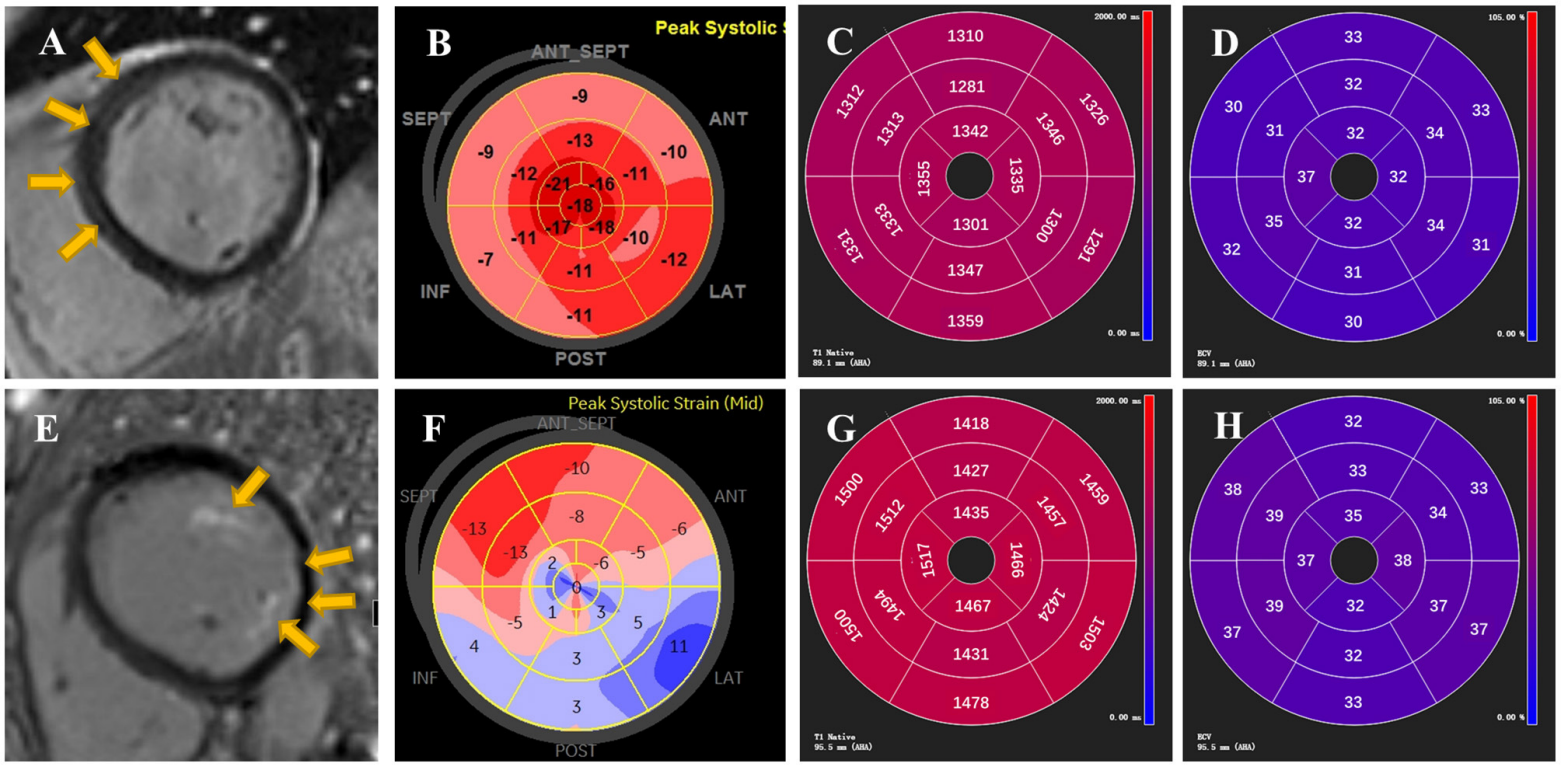
Representative examples of LGE, GLS, native T1 and ECV from two patients with AIDs. On top are demonstrated STE and CMR images of a 44-year-old female patient with tLGEs = 3. LGE showed linear mid-wall enhancement in the septum **(A)** and Bull's Eye image from STE presented mild impairment of longitudinal strain in septal longitudinal strain **(B)**. Global native T1 and ECV were 1,324 ± 23 ms and 32 ± 2%, respectively **(C,D)**. On bottom are reported images from a 51-year-old female patient with tLGEs = 7. CMR showed presence of LGE in left ventricular papillary muscle and lateral wall with a subendocardial pattern **(E)**. An evident decrease of longitudinal strain in Bull's Eye image was found in the free wall and apex **(F)**. Global native T1 and ECV were 1,468 ± 33 ms and 35 ± 3%, respectively **(G,H)**. Yellow arrows indicate the presence of LGE.

The 102 AIDs patients were classified into 2 subgroups based on the presence or absence of LGE (Table 2). Compared to the patients without LGE, those with LGE not only had worse NYHA function (*p* = 0.025) and higher CTnI levels (*p* = 0.045) but also presented marked changes in myocardial deformation, such as lower GLS (*p* = 0.018) and GCS (*p* = 0.021). However, LVEF (*p* = 0.121), diastolic function (*p* = 0.147) and disease duration (*p* = 0.693) did not differ between the above two subgroups. Furthermore, ROC analysis for the detection of LGE presence showed that the cutoff value of GLS was −15% (Supplementary Figure 2), which was used for the classification for high and low GLS. Univariate logistic analysis indicated that both GLS ≥ −15% and CTnI ≥ 0.056 were significantly associated with LGE presence (Table 3). On multivariate logistic analysis, GLS ≥ −15% (OR = 4.98, 95%CI 1.35–18.33, *p* = 0.016) was the only independent predictor of LGE presence when adjusted for age and CTnI. Representative examples of the imaging findings from two patients with LGE are shown in Figure 1. Evident is the consistency between the fibrosis position visualized by LGE and segments with impaired longitudinal strain.

**Table 3:** Univariate and multivariate logistic models for predicting the presence of LGE.

	**Univariate analysis**			**Multivariate analysis**		
	**OR**	**95% CI**	***p***	**OR**	**95% CI**	***p***
Age	1.005	0.978–1.034	0.702	0.996	0.967–1.026	0.799
Disease duration	1.000	0.993–1.007	0.979			
NTproBNP/100	1.025	0.994–1.057	0.116			
CTnI ≥ 0.056	2.813	1.074–7.369	0.035	2.574	0.929–7.130	0.069
LVEF ≥ 50%	4.565	0.985–21.146	0.052			
GLS ≥−15%	5.515	1.525–19.947	0.009	4.979	1.352–18.331	0.016

*Abbreviations as Tables 1, 2*.

**Figure 2:**
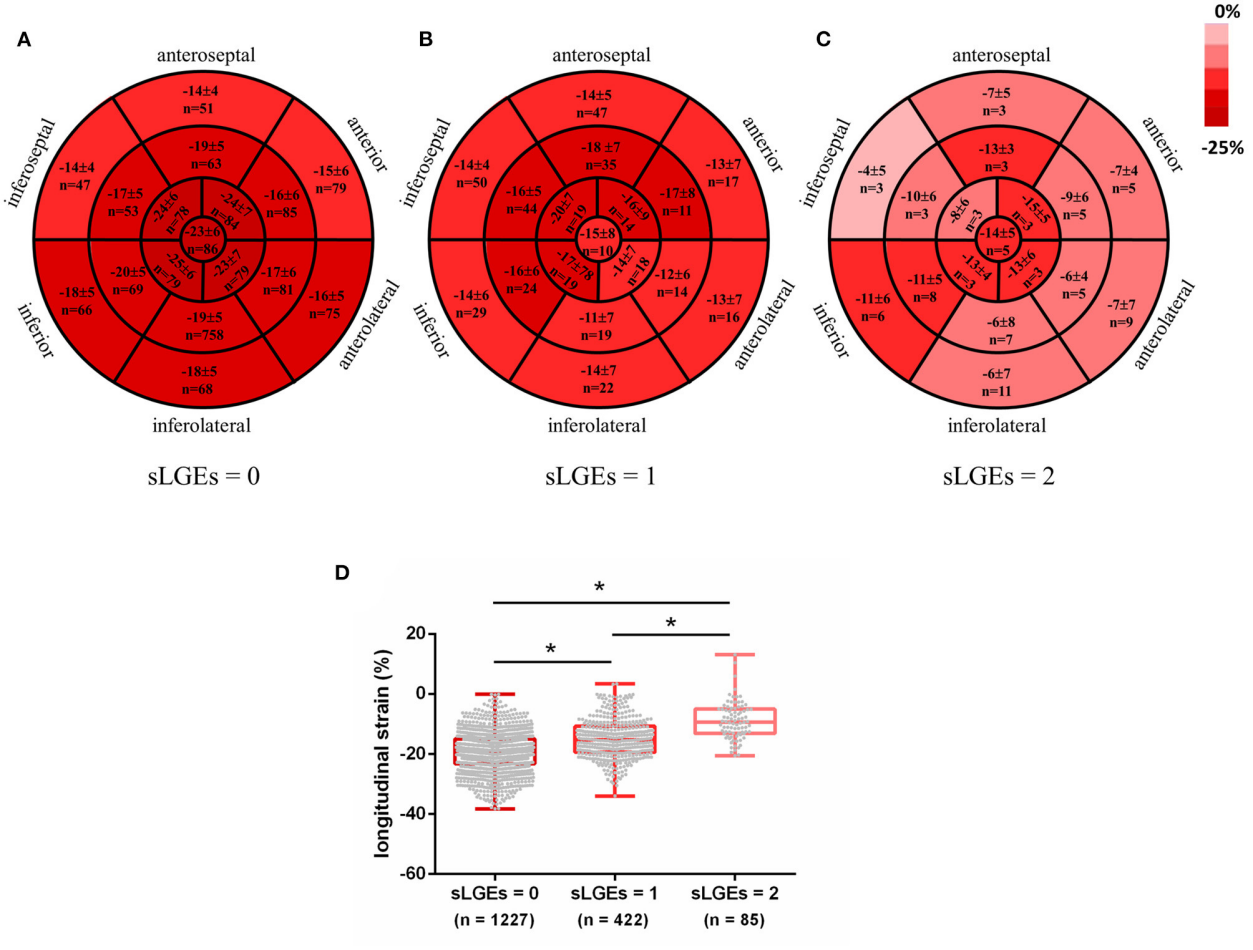
Color coded Bull's Eye Images for longitudinal strain distribution in 17 segments according to LGE involvement extent **(A–C)**. Data expressed as mean ± SD and numbers. Segmental longitudinal strain was ordered decreasingly with increased sLGEs **(D)**. *p < 0.001.

Subsequently, segmental analyses were performed to evaluate whether impaired strain was related to the location and extent of myocardial fibrosis. A total of 1,734 segments were identified in 102 AIDs patients. Each segment was semiquantitated by LGE score: sLGEs = 0 in 1,227 segments, sLGEs = 1 in 422 segments and sLGEs = 2 in 85 segments. Bull's-eye images illustrate the distribution of longitudinal strain in 17 segments according to the extent of LGE involvement (Figure 2). An ordinal decrease was detected in segmental longitudinal strain (−19.3 ± 6.6 vs. −14.9 ± 6.5 vs. −8.9 ± 6.3, p < 0.001) and segmental circumferential strain (−24.8 ± 9.9 vs. −20.5 ± 9.2 vs. −13.4 ± 7.3, p < 0.001) as sLGEs increased (Figure 2 and Supplementary Figure 3). Moreover, segments were categorized by the median native T1 (1,379 ms), as shown in Table 4. Interestingly, almost 40% non-LGE segments presented high native T1 (>1,379 ms). We further found that segmental longitudinal strain was lower (−17.5 ± 6.6 vs. −19.1 ± 6.0, p < 0.001) in non-LGE segments with high native T1 than in those with low native T1. Likewise, a significant impairment of longitudinal strain (−13.2 ± 7.0 vs. −15.5 ± 5.3, *p* < 0.001) was seen in LGE segments with high T1, compared to those with low T1.

**Table 4:** Comparison of strain between segments with high and low native T1 stratified by presence or absence of segmental LGE.

**Variables**	**Native T1 <1,379 ms**	**Native T1 ≥1,379 ms**	***p***
**LGE (–)**	**(*****n*** **=** **590)**	**(*****n*** **=** **394)**	
LS (%)	−19.1 ± 6.0	−17.5 ± 6.6	<0.001
CS (%)	−24.5 ± 10.2	−23.2 ± 9.4	0.033
RS (%)	19.6 ± 13.4	19.0 ± 13.4	0.307
**LGE (+)**	**(*****n*** **=** **131)**	**(*****n*** **=** **303)**	
LS (%)	−15.5 ± 5.3	−13.2 ± 7.0	<0.001
CS (%)	−20.9 ± 9.0	−18.6 ± 9.1	0.015
RS (%)	18.5 ± 12.9	15.9 ± 10.4	0.050

*Abbreviations as Tables 1, 2*.

### Association Between Imaging Findings and Clinical Outcomes

**Figure 3:**
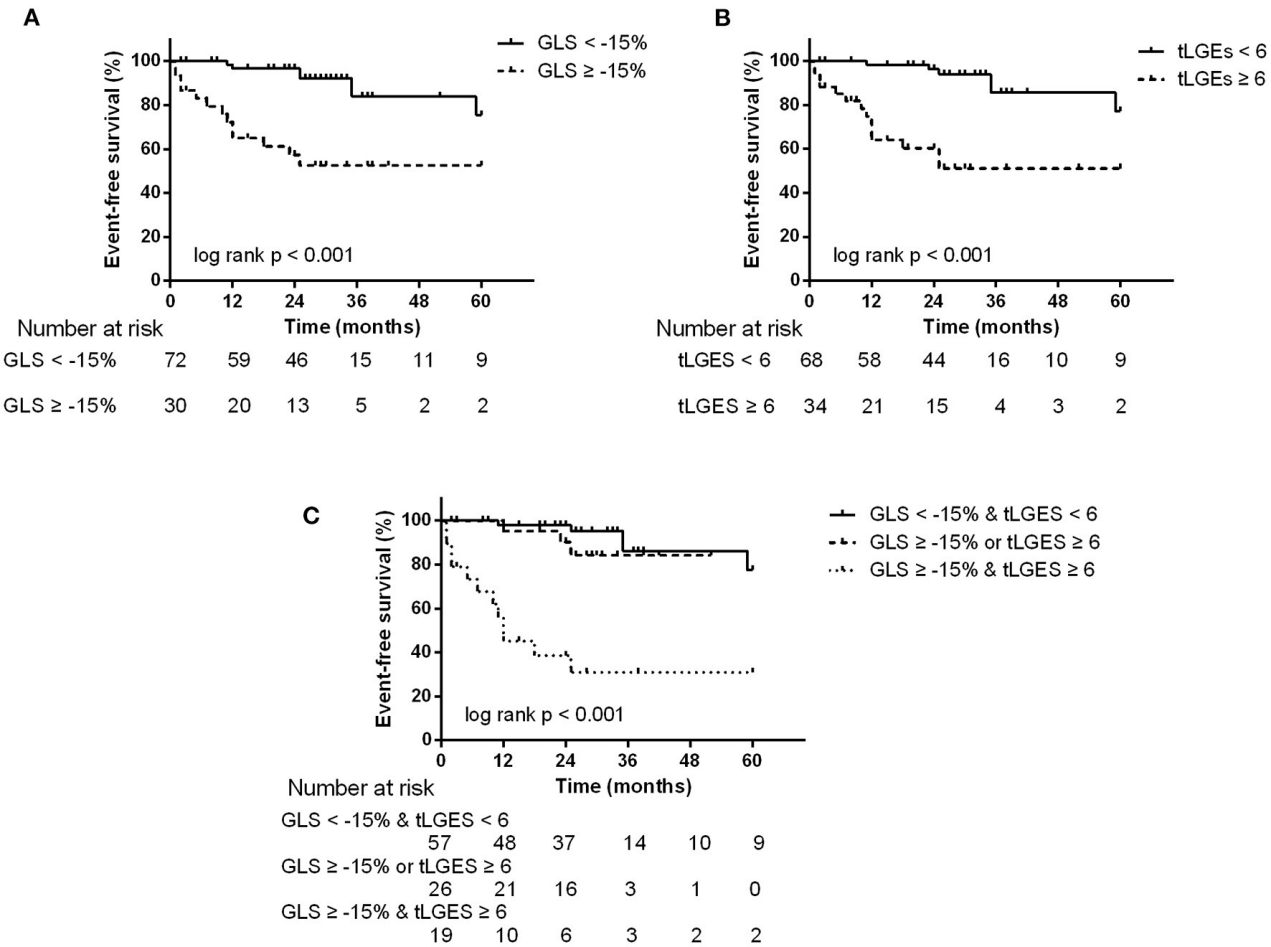
Kaplan-Meier survival curves stratified by abnormal GLS **(A)**, tLGEs **(B)**, and combined GLS and tLGEs **(C)**. Patients with both impaired GLS and high tLGEs had predominantly reduced event-free survival.

During a median follow-up time of 25 months (IQR, 13–35 months), the endpoint occurred in 20 patients: 6 patients died and 14 patients had a cardiovascular event requiring hospitalization (9 heart failure and 5 arrhythmias). By ROC analyses, GLS = −15%, PALS = 15% and tLGEs = 6 were identified as the optimal cutoff values to predict the endpoint (Supplementary Figure 4). Univariate Cox analysis revealed that CTnI ≥ 0.0556, LVEF <50%, GLS ≥ −15%, PALS ≤ 15% and tLGEs ≥ 6 were significantly associated with an increased risk of the primary endpoint. On multivariate analysis, GLS ≥ −15% (HR 3.56, 95%CI 1.28–9.86, *p* = 0.015) and tLGEs ≥ 6 (HR 4.13, 95%CI 1.43–11.92, *p* = 0.009) were still independent risk predictors of the composite outcome (Table 5). Moreover, Kaplan-Meier curves showed that patients with both impaired GLS and high tLGEs were most likely to have predominantly shorter event-free survival (Figure 3).

**Table 5:** Univariate and multivariate Cox regression analyses for predictors of the primary endpoint.

	**Univariate analysis**			**Multivariate analysis**		
	**HR**	**95% CI**	***p***	**HR**	**95% CI**	***p***
Age	1.022	0.995–1.050	0.113			0.412
Age at diagnosis of AIDs	1.016	0.989–1.044	0.241			
Sex	0.501	0.167–1.505	0.218			
Disease duration	1.005	0.998–1.011	0.141			
NTproBNP/100	1.011	0.997–1.025	0.118			
CTnI ≥ 0.056	3.136	1.264–7.777	0.014			0.468
LVEF <50%	4.315	1.758–10.593	0.001			0.713
GLS ≥-15%	5.861	2.329–14.750	<0.001	3.556	1.283–9.856	0.015
PALS ≤ 15%	4.143	1.622–10.587	0.003			0.099
LGE presence	4.287	0.993–18.509	0.051			
tLGEs ≥6	6.952	2.644–18.285	<0.001	4.132	1.432–11.916	0.009

*CI, confidence interval; HR, hard ratio. Other abbreviations as Tables 1, 2*.

### Reproducibility of Imaging Measurements

The intraclass correlation coefficient was used to assess inter- and intraobserver reproducibility. The intra- and interobserver reliability was 0.96 and 0.89 for GLS, respectively, and the interobserver correlation coefficient was 0.99 and 0.92 for native T1 and ECV, respectively, suggesting high reproducibility of imaging measurements in our study.

## Discussion

In the present study, we used non-invasive multimodal imaging to evaluate myocardial fibrosis and predict clinical outcomes in AIDs patients. The main findings are as follows: First, a high prevalence of myocardial fibrosis as demonstrated by CMR was identified in AIDs patients with clinically suspected cardiac involvement. Second, impaired longitudinal strain had the potential to reflect the presence and extent of LGE and to sensitively detect abnormally increased T1 value. Third, both GLS ≥ −15% and tLGEs ≥ 6 were independently associated with the primary endpoint and improved the risk stratification for prognosis in AIDs patients.

### Strain Reflects Myocardial Fibrosis

Previous studies have shown that LGE is a reference standard used to detect focal myocardial fibrosis in AIDs in a timely manner (26). In the current study, we found that GLS could be used as an independent predictor of the presence of LGE in AIDs patients. Indeed, myocardial deformation has been applied to detect underlying fibrosis in other clinical conditions (27). For instance, a recent study by Raucci et al. showed that GLS can strongly predict LGE burden in Duchenne muscular dystrophy (28). Abnormal collagen deposition and matrix remodeling lead to changes in movement of myocardial fibers, which further affects myocardial deformation. This suggests impaired strain can reflect the functional consequence of interstitial myocardial fibrosis to a certain extent (29). We further demonstrated an ordinal decrease in segmental longitudinal shortening with increased sLGEs, which underscores that the severity of strain impairment is dependent on the magnitude of myocardial fibrosis, leading to incremental dysfunction in involved segments. In some diseases, strain is compromised in segments with LGE, suggesting a more advanced stage of myocardial involvement (27). Therefore, regional longitudinal strain had the potential to identify specific location and stratify different extent of focal myocardial fibrosis.

Interestingly, patients with LGE had not only lower GLS but also lower GCS compared to those without LGE. Longitudinal strain is mainly governed by subendocardial fibers and is the most vulnerable deformation of ventricular mechanics (9, 30). Therefore, GLS, as a sensitive indicator to reflect the presence of myocardial impairment, has been widely used to detect subclinical cardiac dysfunction. Whereas, GCS is predominantly governed by subepicardial myofibers and remains relatively sparing or presents exaggerated compensation in early stage of disease. The simultaneous attenuation of GLS and GCS results from concomitant subendocardial and subepicardial involvement, indicating a progressive myocardial disease with transmural impairment (9, 30). This is consistent with our findings that patients with LGE presented both impaired longitudinal and circumferential deformation, highlighting loss of GLS and GCS was dependent on the severity of myocardial involvement evaluated by the presence and degree of LGE. Accordingly, the assessment of myocardial mechanics may help to provide pathophysiologic insight into the mechanism of cardiac involvement in AIDs.

T1 mapping allows the discrimination and quantification of diffuse myocardial fibrosis missed by LGE (1). Native T1 and ECV have been proven to have a good correlation with histological fibrosis in other clinical contexts (31). Our data strengthened the evidence that the presence of diffuse myocardial fibrosis in AIDs patients manifested by elevated native T1 and expanded ECV. Furthermore, abnormally high T1 value was found in almost 40% of non-LGE segments, highlighting that native T1 might be an early indicator to detect myocardial injury before the development of irreversible fibrosis and functional decompensation (32, 33). Notably, segmental longitudinal strain could sensitively and significantly discriminate abnormalities in native T1 value even in non-LGE segments. This suggests that the decrease in longitudinal strain has the potential to detect hidden myocardial impairment in normal appearing myocardium as evaluated by LGE.

### Myocardial Strain and Fibrosis as Outcome Predictors

To date, the role of imaging findings in portending the risk of adverse clinical outcomes in AIDs patients has not been well-studied (5). Our research showed that impaired strain and tLGEs not only indicated the cardiac performance of the disease-associated pathophysiologic mechanisms but also acted as independent risk factors for the composite endpoint. A close relationship between GLS and poor clinical events in patients with SLE and rheumatoid arthritis was confirmed in recent studies (20, 34), supporting our results that patients with abnormal GLS had worse event-free survival than those with normal GLS. LGE has been reported to be a strong predictor of adverse clinical events in non-ischemic cardiomyopathy, whereas the absence of LGE cannot ensure freedom from poor outcomes (35). Our findings demonstrated that the extent of LGE is likely to be more valuable for predicting prognosis than is the presence of LGE alone. Moreover, the addition of GLS to tLGEs contributed to marked improvement in the discriminative ability to identify high-risk patients.

Additionally, we noticed that decreased PALS was a potential risk factor for the endpoint, but no longer a significantly independent predictor after multivariate adjustment. Recently, the role of PALS in disease progression and adverse outcomes has aroused much interest. PALS can detect LV diastolic dysfunction and correlate well with LV filling pressure even with normal LA size (36, 37). In rheumatoid arthritis, impaired PALS may precede abnormal LV performance and be an early indicator of reversible cardiac dysfunction (38). Indeed, PALS was found to be markedly lower in AIDs patients according to our data. Therefore, the clinical significance and prognostic value of decreased PALS in AIDs deserves more investigation in future work.

To the best of our knowledge, this is the first study to apply multimodal imaging to the assessment of clinical prognosis in patients with AIDs. The results highlighted that combination of STE and CMR imaging is conducive to identifying AIDs patients at high risk for adverse clinical events.

### Clinical Implications

AIDs patients with clinically suspected cardiac involvement presented high prevalence of myocardial fibrosis and experienced more cardiovascular events, highlighting that this patient population should be given enough attention, prompt evaluation and timely treatment. Both STE and CMR can provide important information to illuminate myocardial involvement and reveal prognosis. STE is the first line imaging method to detect myocardial impairment due to its more affordable and easily accessible characteristics. Given the relationship of GLS and CMR indicators of fibrosis, impaired longitudinal strain indicates the presence of myocardial injury and requires due attention. Then, CMR examination is needed to further identify more precise localization and the extent of myocardial involvement. It is not an alternative but a complementary relationship among various imaging modalities (39). The complementary strengths of STE and CMR will contribute to guiding risk stratification and cardiac treatment for AIDs patients.

### Limitations

We recognize that our study has several limitations. First, this was a single-center cohort study with a relatively small sample size, so the validation of our findings should be warranted in multi-center cohorts. Second, the etiological heterogeneity of the study population confers some limitations to our research. Nevertheless, myocardial fibrosis is a common feature on cardiac pathology in patients with SLE (6), IIM (40), SS (41), and SSc (2). The shared pathophysiologic mechanisms of myocardial involvement in AIDs, such as immune complex deposition and chronic cumulative inflammation, may account for the commonality across imaging findings. Moreover, no difference was found in the comparison of imaging parameters and adverse events among the four types of AIDs. Third, our study only enrolled AIDs patients with clinically suspected cardiac involvement, so extrapolation of our results to the entire population with AIDs needs to be validated. Fourth, we failed to distinguish acute and chronic myocardial lesions due to no assessment of myocardial edema by T2 mapping. Finally, the low event rate during follow-up may increase the uncertainty of the multivariate Cox analysis and limit further analysis of secondary endpoints. Therefore, large cohorts with long follow-up periods are warranted in future studies to confirm our results.

## Conclusions

In AIDs patients, myocardial strain derived from STE had the potential to reflect the presence and extent of myocardial fibrosis. The combination of GLS and tLGEs contributed to predicting prognosis and improving the risk stratification. These findings highlight the clinical importance of non-invasive multimodal imaging in assessing myocardial involvement and adverse outcomes.

## Data Availability Statement

The original contributions presented in the study are included in the article/Supplementary Material, further inquiries can be directed to the corresponding author/s.

## Ethics Statement

The studies involving human participants were reviewed and approved by the Institutional Review Board of Peking Union Medical College Hospital. The patients/participants provided their written informed consent to participate in this study. Written informed consent was obtained from the individual(s) for the publication of any potentially identifiable images or data included in this article.

## Author Contributions

WC and YW are the guarantors of the integrity of the entire study. FJ, XL, HY, WC, and YW are responsible for the study concepts and design. FJ, XL, SJ, JY, XF, YaZ, YL, YuZ, JL, and LF performed the experimental studies. FJ, XL, DZ, WC, and HY analyzed and interpreted the data. FJ and DZ performed the statistical analysis and all the results were checked by YaZ, YL, YuZ, and JL. FJ, XL, SJ, JY, and XF prepared the first draft of the manuscript, which was critically revised by WC, YW, DZ, HY, and LF. All authors approved the final version to be published.

## Funding

This work was supported by the Beijing Natural Science Foundation (7192156), the Capital's Funds for Health Improvement and Research, CFH (2020-2-40110), and the CAMS Innovation Fund for Medical Sciences (CIFMS, 2020-I2M-C&T-B-006) to WC.

## Conflict of Interest

The authors declare that the research was conducted in the absence of any commercial or financial relationships that could be construed as a potential conflict of interest.

## Publisher's Note

All claims expressed in this article are solely those of the authors and do not necessarily represent those of their affiliated organizations, or those of the publisher, the editors and the reviewers. Any product that may be evaluated in this article, or claim that may be made by its manufacturer, is not guaranteed or endorsed by the publisher.
