# Supplementary material for: Predictive Value of Echocardiographic Strain for Myocardial Fibrosis and Adverse Outcomes in Autoimmune Diseases
Source: Front Cardiovasc Med. 2022 Feb 21;9:836942. doi: 10.3389/fcvm.2022.836942 (PMC8899104; doi:10.3389/fcvm.2022.836942)
Supplement: Supplementary file 1 [file Data_Sheet_1.docx]

Supplementary Material

# Supplementary Table

## Table S1. Comparison of baseline cardiovascular characteristics and follow-up parameters according to classification of AIDs.

| Variables | SLE (n = 48) | IIM (n = 38) | SS (n = 5) | SSc (n = 11) | p |
| --- | --- | --- | --- | --- | --- |
| **Clinically suspected cardiac involvement [n (%)]** |  |  |  |  |  |
| Symptoms | 11 (23%) | 14 (37%) | 1 (20%) | 4 (36%) | 0.483 |
| NYHA class ≥ 2 | 9 (19%) | 11 (29%) | 1 (20%) | 4 (36%) | 0.541 |
| ECG abnormalities | 17 (35%) | 21 (55%) | 2 (40%) | 7 (64%) | 0.177 |
| CTnI elevation (> 0.056ug/L) | 8 (17%) | 23 (61%) | 1 (20%) | 7 (64%) | < 0.001 |
| **Comorbidity [n (%)]** |  |  |  |  |  |
| Hypertension | 9 (19%) | 10 (26%) | 2 (40%) | 2 (18%) | 0.684 |
| Diabetes | 0 | 2 (5%) | 0 | 1 (9%) | 0.298 |
| CHD | 2 (4%) | 8 (21%) | 1 (20%) | 1 (9%) | 0.117 |
| **Echocardiography** |  |  |  |  |  |
| LVEF (%) | 62.0 ± 10.0 | 60.5 ± 12.8 | 58.4 ± 23.0 | 56.5 ± 17.4 | 0.926 |
| LVEDVi (ml/m2) | 51.6 ± 16.0 | 57.4 ± 20.2 | 62.0 ± 11.9 | 53.3 ± 20.0 | 0.302 |
| LVESVi (ml/m2) | 21.0 ± 11.6 | 23.2 ± 12.9 | 28.2 ± 13.1 | 25.2 ± 18.8 | 0.716 |
| E/A ratio | 1.2 ± 0.4 | 1.1 ± 0.3 | 1.1 ± 0.6 | 1.3 ± 0.5 | 0.457 |
| Lateral e’ (cm/s) | 10.3 ± 4.4 | 9.2 ± 2.7 | 9.6 ± 4.0 | 8.4 ± 4.2 | 0.411 |
| Septal e’ (cm/s) | 7.3 ± 2.8 | 7.1 ± 2.4 | 5.0 ± 1.6 | 5.5 ± 2.2 | 0.073 |
| E/e’ | 10.6 ± 6.9 | 10.1 ± 3.4 | 12.8 ± 4.7 | 15.5 ± 8.0 | 0.068 |
| TRV (m/s) | 2.8 ± 0.8 | 2.4 ± 0.5 | 2.9 ± 1.0 | 2.8 ± 0.6 | 0.211 |
| LAVi (ml/m^2^) | 31.2 ± 14.4 | 35.0 ± 12.7 | 33.5 ± 11.5 | 37.9 ± 17.3 | 0.299 |
| PASP (mmHg) | 38.7 ± 22.6 | 28.8 ± 13.1 | 41.3 ± 28.1 | 36.4 ± 15.0 | 0.108 |
| Diastolic dysfunction [n (%)] | 8 (17%) | 12 (32%) | 1 (20%) | 6 (55%) | 0.059 |
| GLS (%) | -18.4 ± 4.4 | -18.0 ± 5.3 | -17.1 ± 5.2 | -14.6 ± 7.8 | 0.671 |
| GCS (%) | -23.7 ± 5.5 | -24.2 ± 7.8 | -23.1 ± 7.2 | -20.5 ± 7.7 | 0.568 |
| GRS (%) | 18.1 ± 5.4 | 17.4 ± 6.5 | 23.6 ± 8.6 | 18.7 ± 9.7 | 0.423 |
| **CMR** |  |  |  |  |  |
| LGE (+) | 33 (69%) | 26 (68%) | 4 (80%) | 8 (73%) | 0.951 |
| Septal | 13 (27%) | 5 (13%) | 1 (20%) | 2 (18%) | 0.109 |
| Free-wall | 4 (8%) | 0 (0%) | 1 (20%) | 2 (18%) |  |
| Both | 16 (33%) | 21 (55 | 2 (40%) | 4 (36%) |  |
| tLGE score | 3 (0, 5) | 4 (0, 11.5) | 5 (1.5, 18.5) | 4 (0, 21) | 0.290 |
| Native T1 (ms) | 1404 ± 73 | 1369 ± 78 | 1427 ± 66 | 1392 ± 68 | 0.099 |
| ECV (%) | 32.9 ± 5.8 | 33.4 ± 6.6 | 35.5 ± 4.0 | 32.8 ± 5.5 | 0.685 |
| **Follow-up** |  |  |  |  |  |
| Time (month) [median (IQR)] | 29 (22−38) | 23 (9−32) | 29 (2−60) | 19 (11−27) | 0.071 |
| The primary endpoint [n (%)] | 6 (13%) | 8 (21%) | 1 (20%) | 5 (45%) | 0.100 |

AIDs, autoimmune diseases; CMR, cardiovascular magnetic resonance; CTnI, cardiac troponin I; ECG, electrocardiogram; ESV, end-systolic volume; EF, ejection fraction; GCS, global circumferential strain; GLS, global longitudinal strain; GRS, global radial strain; IIM, idiopathic inflammatory myopathy; IQR, interquartile range; LAVi, left atrium volume index; LGE, late gadolinium enhancement; LV, left ventricular; PALS, peak left atrial longitudinal strain; PASP, pulmonary artery systolic pressure; SLE, Systemic lupus erythematosus; SS, Sjogren’s syndrome; SSc, Systemic scleroderma; tLGEs, total LGE score; TRV, tricuspid regurgitation velocity.

## Table S2. Comparison of baseline cardiovascular characteristics and follow-up parameters according to disease activity.

| Variables | Active patients  (n = 48) | Remission patients  (n = 54) | p |
| --- | --- | --- | --- |
| **Clinically suspected cardiac involvement [n (%)]** |  |  |  |
| Symptoms | 16 (33%) | 14 (26%) | 0.515 |
| NYHA class ≥ 2 | 12 (25%) | 13 (24%) | 1.000 |
| ECG abnormalities | 23 (48%) | 24 (44%) | 0.843 |
| CTnI elevation (> 0.056ug/L) | 18 (38%) | 21 (39%) | 1.000 |
| **Comorbidity [n (%)]** |  |  |  |
| Hypertension | 11 (23%) | 12 (22%) | 1.000 |
| Diabetes | 0 (0%) | 3 (5%) | 0.243 |
| CHD | 4 (8%) | 8 (15%) | 0.362 |
| **Echocardiography** |  |  |  |
| LVEF (%) | 59.6 ± 13.3 | 61.3 ± 13.0 | 0.385 |
| LVEDVi (ml/m2) | 58.3 ± 17.4 | 51.4 ± 18.2 | 0.023 |
| LVESVi (ml/m2) | 24.5 ± 12.5 | 21.1 ± 13.5 | 0.047 |
| E/A ratio | 1.3 ± 0.4 | 1.1 ± 0.4 | 0.051 |
| Lateral e’ (cm/s) | 10.2 ± 4.1 | 9.2 ± 3.7 | 0.361 |
| Septal e’ (cm/s) | 7.3 ± 2.7 | 6.6 ± 2.7 | 0.210 |
| E/e’ | 10.9 ± 6.7 | 11.1 ± 5.4 | 0.546 |
| TRV (m/s) | 2.6 ± 0.6 | 2.7 ± 0.8 | 0.394 |
| LAVi (ml/m^2^) | 35.0 ± 13.9 | 32.0 ± 14.2 | 0.255 |
| PASP (mmHg) | 32.2 ± 17.1 | 37.1 ± 21.6 | 0.459 |
| Diastolic dysfunction [n (%)] | 10 (21%) | 17 (31%) | 0.265 |
| GLS (%) | -17.3 ± 5.2 | -18.4 ± 5.0 | 0.215 |
| GCS (%) | -22.7 ± 5.8 | -24.3 ± 7.5 | 0.251 |
| GRS (%) | 18.8 ± 6.9 | 17.7 ± 6.2 | 0.603 |
| **CMR** |  |  |  |
| LGE (+) | 30 (63%) | 41 (76%) | 0.196 |
| tLGE score | 3 (0, 6) | 4 (0.8, 11) | 0.132 |
| Native T1 (ms) | 1395 ± 87 | 1384 ± 64 | 0.701 |
| ECV (%) | 33.6 ± 5.8 | 32.9 ± 6.2 | 0.406 |
| **Follow-up** |  |  |  |
| Time (month) [median (IQR)] | 25 (13−35) | 25 (12−35) | 0.901 |
| The primary endpoint [n (%)] | 9 (19%) | 11 (20%) | 1.000 |

Abbreviations as Table S1.

# Supplementary Figures

## Supplementary Figure S1

**
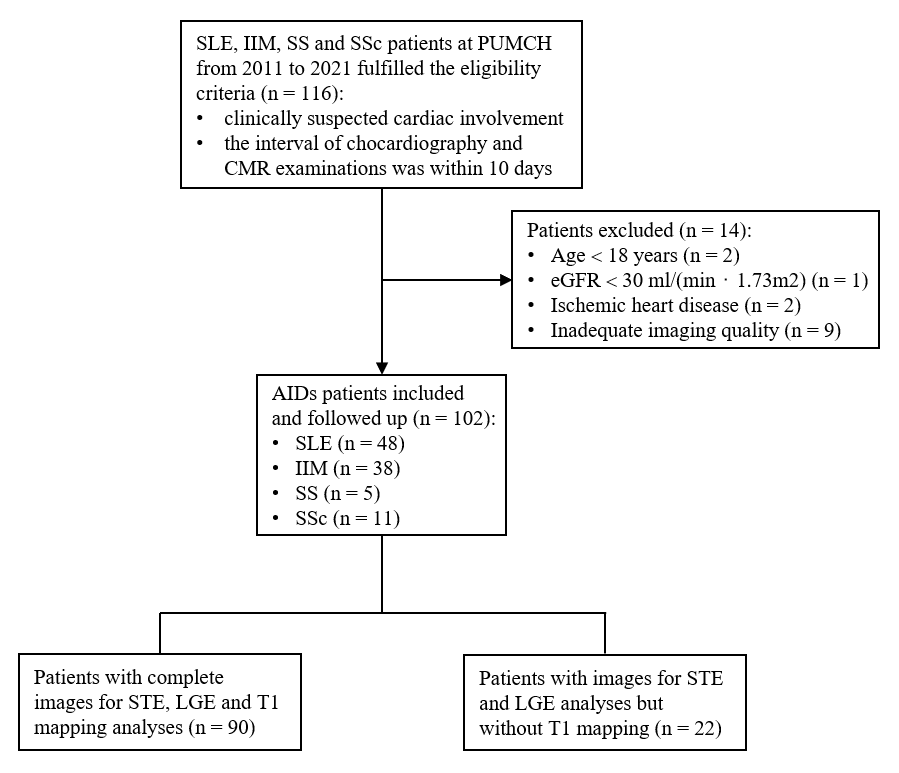
**

**Figure S1.** Flowchart of patients screening. AIDs, autoimmune diseases; CMR, cardiovascular magnetic resonance; eGFR, estimated glomerular filtration rate; IIM, idiopathic inflammatory myopathy; LGE, late gadolinium enhancement; PUMCH, Peking Union Medical College Hospital; SLE, systemic lupus erythematosus; SS, Sjogren’s syndrome; SSc, systemic scleroderma; STE, speckle-tracking echocardiography.

## Supplementary Figure S2


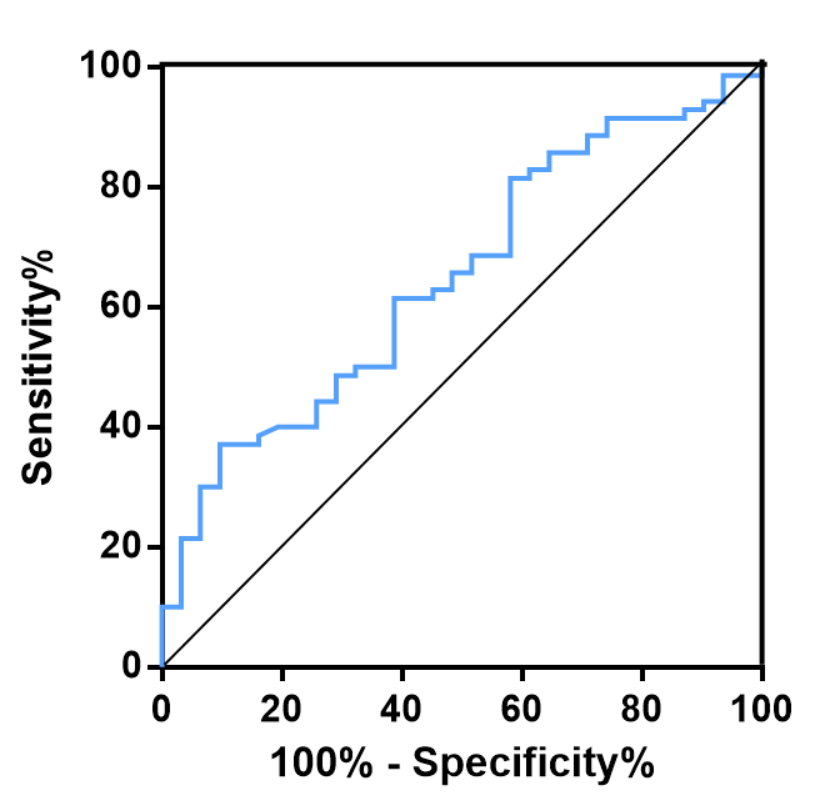


**Figure S2.** Receiver Operator Curve for GLS to detect the presence of LGE. The area under the curve was 0.65 (95% CI 0.54-0.76, p = 0.018), and the sensitivity and specificity for GLS = 15% were 37% and 90%, respectively.

## Supplementary Figure S3


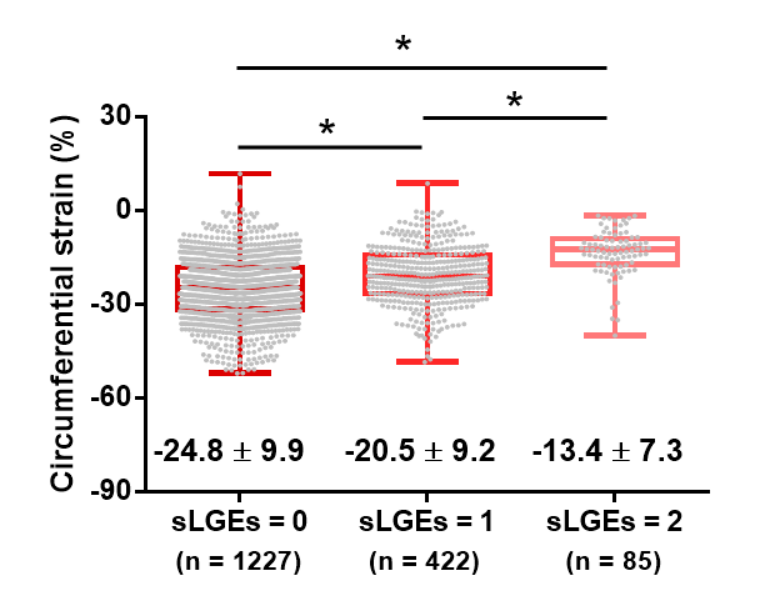


**Figure S3.** Segmental circumferential strain was ordered decreasingly with increased sLGEs.

## Supplementary Figure S4


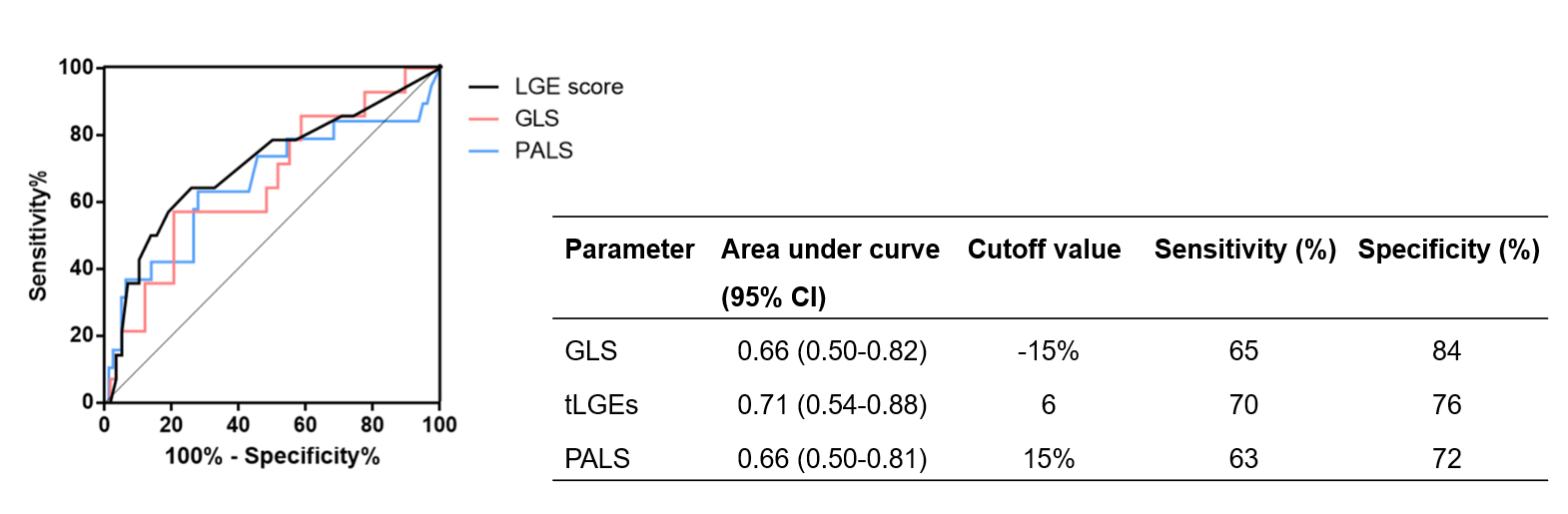


**Figure S4.** Receiver Operator Curve for GLS, LGE score and PALS to predict the primary endpoint.
